# Mucin Gene Deficiency in Mice Impairs Host Resistance to an Enteric Parasitic Infection

**DOI:** 10.1053/j.gastro.2010.01.045

**Published:** 2010-05

**Authors:** Sumaira Z. Hasnain, Huaqing Wang, Jean–Eric Ghia, Nihal Haq, Yikang Deng, Anna Velcich, Richard K. Grencis, David J. Thornton, Waliul I. Khan

**Affiliations:** ⁎ Faculty of Life Sciences, University of Manchester, Manchester, United Kingdom; ‡ Farncombe Family Digestive Health Research Institute, Department of Pathology & Molecular Medicine, McMaster University, Hamilton, Ontario, Canada; § Department of Oncology, Albert Einstein Cancer Center/Montefiore Medical Center, Bronx, New York

**Keywords:** Muc2, Goblet Cell, Enteric Infection, Host Resistance, Innate Immunity, ATP, adenosine triphosphate, BrdU, bromodeoxyuridine, IL-4, interleukin-4, KO, knockout, mMuc2, murine Muc2, PAS, periodic acid Schiff, Relm, resistin-like molecule, RT-PCR, reverse transcription–polymerase chain reaction, SCID, severe combined immunodeficient, Tff3, trefoil factor 3, T_H_, T helper, WT, wild-type

## Abstract

**Background & Aims:**

Hyperplasia of mucin-secreting intestinal goblet cells accompanies a number of enteric infections, including infections by nematode parasites. Nevertheless, the precise role of mucins in host defense in nematode infection is not known. We investigated the role of the mucin (Muc2) in worm expulsion and host immunity in a model of nematode infection.

**Methods:**

Resistant (BALB/c, C57BL/6), susceptible (AKR), and Muc2-deficient mouse strains were infected with the nematode, *Trichuris muris*, and worm expulsion, energy status of the whipworms, changes in mucus/mucins, and inflammatory and immune responses were investigated after infection.

**Results:**

The increase in Muc2 production, observed exclusively in resistant mice, correlated with worm expulsion. Moreover, expulsion of the worms from the intestine was significantly delayed in the Muc2-deficient mice. Although a marked impairment in the development of periodic acid Schiff (PAS)–stained intestinal goblet cells was observed in Muc2-deficient mice, as infection progressed a significant increase in the number of PAS-positive goblet cells was observed in these mice. Surprisingly, an increase in Muc5ac, a mucin normally expressed in the airways and stomach, was observed after infection of only the resistant animals. Overall, the mucus barrier in the resistant mice was less permeable than that of susceptible mice. Furthermore, the worms isolated from the resistant mice had a lower energy status.

**Conclusions:**

Mucins are an important component of innate defense in enteric infection; this is the first demonstration of the important functional contribution of mucins to host protection from nematode infection.

The mucus barrier is an essential part of the innate immune system which hydrates and protects the underlying epithelia. The gel-like properties of the barrier are mainly due to the polymeric mucins that are the main secretory products of epithelial goblet cells.^1–3^ The colonic epithelium expresses mainly MUC2/Muc2 in large amounts which is stored in bulky apical granules of the goblet cells and is the most important factor determining the goblet cell morphology.^4–6^ Muc2 forms a heterogeneous mucus barrier that is proposed to contain 2 distinct layers; a “loose” outer layer that bacteria can penetrate and an adherent inner layer that excludes bacteria from direct contact with the underlying epithelia.^7^

Alterations or absence of MUC2 production can lead to many common human disorders such as colon carcinoma,^8^ ulcerative colitis,^9^ and celiac disease.^10^ A role for Muc2 in the suppression of colorectal carcinoma has also been suggested because Muc2 knockout (KO) mice spontaneously develop colitis and adenomas that progress to invasive adenocarcinoma,^11^ suggesting an important function for this mucin in colonic protection.^6^ Furthermore, missense mutations in the *Muc2* gene results in aberrant Muc2 oligomerization, leading to endoplasmic reticulum stress and subsequently increased susceptibility to colitis.^12^

Hyperplasia of mucin-producing goblet cells has been described in a number of parasitic infections, including *Nippostrongylus brasiliensis, Hymenolepis diminuta*, *Trichinella spiralis*, and *Trichuris muris*.^13–17^ Putative mechanisms underlying the protective role of mucins against infectious agents include the demonstration of trapping of *Hymenolepis diminuta*^17^ and *Trichinella spiralis*^18^ in the mucus and inhibition of parasite motility and feeding capacity.^18–20^ Goblet cell response, in all 4 of these nematode models, is thought to be under the control of a T helper (T_H_) 2-type immune response and is considered as a potential effector mechanism.^21–23^ A number of goblet cell bioactive factors such as resistin-like molecule-β (Relm-β), intelectin, and calcium-activated chloride channel-3 have been suggested to play an important role in nematode infection.^24,25^ However, a definitive and precise role of mucins, the main secreted product of goblet cells, in host defense in intestinal nematode infection remains to be elucidated.

The nematode *T muris* inhabits the cecum of mice and is closely related at the morphologic, physiologic, and antigenic levels to *Trichuris trichuria*, the causative agent of chronic trichuriasis in human beings.^26^ In this parasitic infection, strains resistant to chronic infection (BALB/c, C57BL/6) expel the parasites through the generation of a T_H_2-type immune response, whereas susceptible strains (AKR), which do not expel the worms, develop a T_H_1-type immune response.^22,27^ In this study, we demonstrated that the increase in Muc2, the main determinant of mucus barrier properties, correlates with worm expulsion. In the absence of Muc2 there is a delay in worm expulsion, but interestingly Muc5ac is up-regulated in the Muc2-deficient mice before expulsion. Moreover, Muc5ac is up-regulated in the wild-type (WT) mice that are resistant to infection, but not in those unable to expel. The physical properties of the mucus barrier are also altered during infection, resulting in a less-porous network, with overall changes having a direct effect on the viability of the whipworm. Collectively, these data show for the first time a protective role for mucins in nematode infection.

## Materials and Methods

### Animals

Breeding pairs of Muc2-KO mice originally produced by gene mutation^11^ and their WT (C57BL/6) littermates (Albert Einstein Medical College, New York, NY) were kept at the animal facilities of McMaster University (Hamilton, ON, Canada). AKR, BALB/c (Harlan, UK), and severe combined immunodeficient (SCID) mice were maintained in the Biological Services Unit at Manchester University. The protocols used were in accordance with guidelines by the McMaster University Animal Care Committee, Canadian Council on the Use of Laboratory Animals, and the Home Office Scientific Procedures Act (1986). All mice were kept in sterilized, filter-topped cages, and fed autoclaved food in the animal facilities. Only 6- to 10-week-old male mice were used.

### Parasitologic Techniques

The techniques used for *T muris* maintenance and infection were described previously.^28^ Mice were orally infected with approximately 100–300 eggs for a high-dose infection and <15 eggs for a low-dose infection. Worm burdens were assessed by counting the number of worms present in the cecum as described previously.^28^

### Histology, Immunohistochemistry, and Immunofluorescence

A 1-cm segment or the whole cecum (rolled) was fixed in 10% neutral buffered formalin or 95% ethanol and processed with the use of standard histologic techniques. Sections were treated with 0.1 mol/L KOH for 30 minutes before staining with periodic acid Schiff (PAS) reaction.^29^ Slides were counterstained with either H&E or 1% fast-green. Standard immunohistochemical and immunofluorescent staining methods^29,30^ were used to determine the levels of Muc2, Muc5ac, Relm-β, and trefoil factor 3 (Tff3).

### Antibodies

Immunodetection was carried out with the use of a polyclonal antibody raised against a murine Muc2 (mMuc2).^12^ Commercially available 45M1 antibody was used for the detection of mouse Muc5ac.^31^ The mouse Muc5b-specific antibody^32^ was a kind gift from Dr Camille Ehre (University of North Carolina, Chapel Hill). Commercially available mRelm-β (Abcam, Cambridge, UK) and mITF (Santa Cruz Biotechnology Inc, Santa Cruz, CA) antibodies were used to detect Relm-β and Tff3, respectively. Detection of bromodeoxyuridine (BrdU) incorporated into nuclei was carried out with the use of a monoclonal anti-BrdU antibody (AbD Serotec, Oxford, UK).^33^

### Mucus Extraction and Agarose Gel Electrophoresis

The cecum was gently flushed with phosphate-buffered saline and scraped, and mucus was solubilized in 8 mol/L guanidium chloride. Subsequently, extracted mucus samples were reduced with 50 mmol/L dithiothreitol and carboxylmethylated with 0.125 mol/L iodoacetamide before electrophoresis on a 1% (wt/vol) agarose gel. Mucins were detected after Western blotting with mucin-specific antisera.^34^

### Analysis of Mucus Network Properties

Cecal tissue isolated from BALB/c and AKR mice was cut longitudinally, washed with phosphate-buffered saline, and kept hydrated in a 6-well plate. Blue fluorescently labeled polymer microspheres (0.1 μm; Dukes Scientific, Dorchester, United Kingdom) were placed on top of the luminal surface of the cecum (set as a reference) and their position was analyzed with the use of the Nikon (Melville, NY) C1 Upright confocal microscope. Three-dimensional optical stacks were taken every 5 μm and combined to obtain a z-stack at the time points stated.

### Energy Status of Worms

The CellTiter-Glo luminescent cell viability assay (Promega, Madison, WI) was carried out according to manufacturer's instructions. Relative light units were calculated per worm as follows: relative light unit = (sample light units − blank light units)/number of worms. Substrate only was used as a blank control, whereas worms were boiled before homogenization for negative controls. To determine recovery of energy status, worms recovered were washed extensively in Dulbecco's modified Eagle's medium, added to 6-well plates with LS174T cells (maintained as previously described by Hayes et al^35^) for 24 hours before measuring adenosine triphosphate (ATP) levels.

### Statistical Analysis

All results are expressed as the mean ± standard error of the mean. Statistical analysis was performed with the use of SPSS Version 16.0 (SPSS Inc, Chicago, IL). Statistical significance of different groups was assessed with parametric tests (one-way analysis of variance with post test after statistical standards or paired Student *t* test). *P* < .05 was considered statistically significant.

## Results

### Increased Muc2 Production Correlates With Worm Expulsion

**Supplementary Figure 1:**
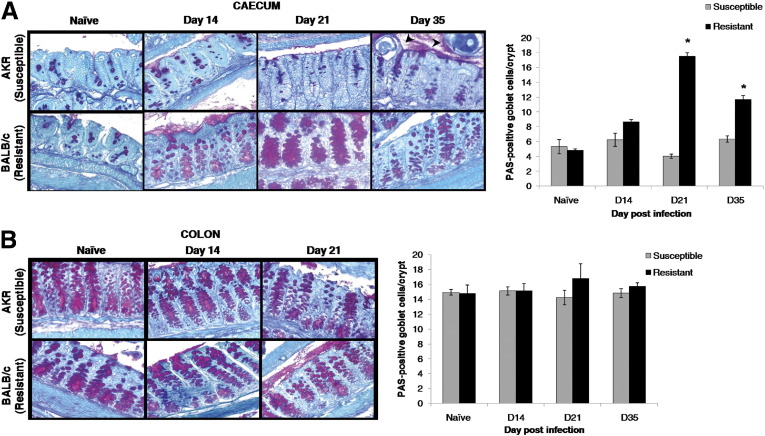
PAS staining in the cecum showed a significant increase in goblet cell numbers only in the resistant (BALB/c) mice with infection (*A*). Worms are highlighted by *arrows* visible in the sections from susceptible mice. No main changes in goblet cell numbers in the colon of resistant (BALB/c) and susceptible (AKR) mice on day 14 and day 21 after infection compared with naïve (*B*). Representative of 3 mice. *P < .05.

**Figure 1:**
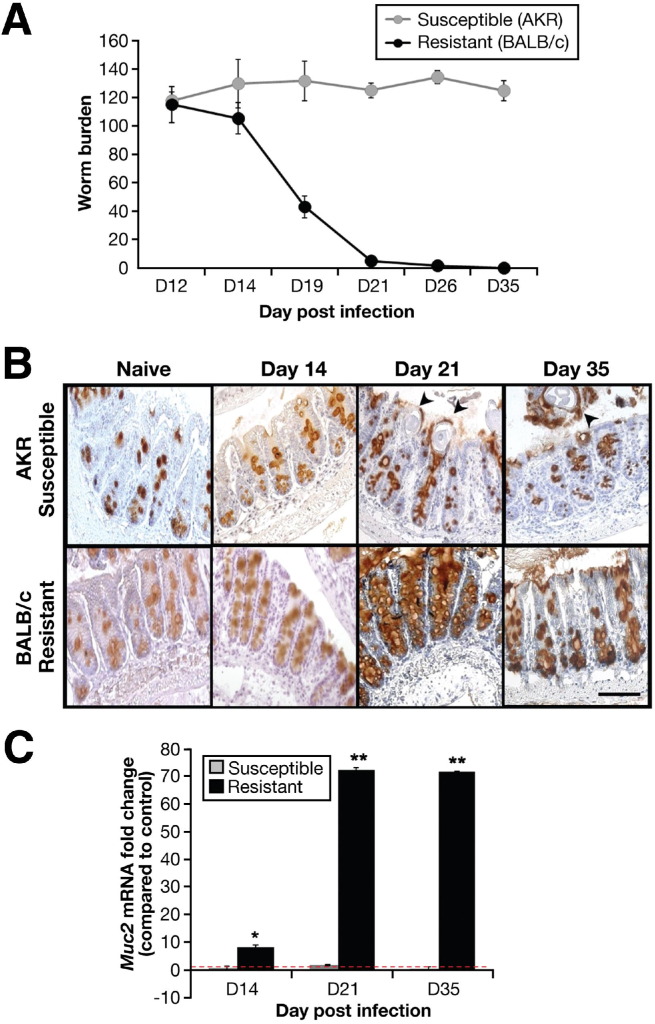
Worm burdens were assessed in both resistant (BALB/c) and susceptible (AKR) mice (*A*). Immunohistochemistry with mMuc2 antibody (*B*) and RT-PCR (*C*) were used to determine changes in Muc2 levels during infection. Nematodes are depicted by *arrows* (B). *Red dashed line* indicates naïve levels (*C*). Representative of 3 mice. Scale bar, 50 μm. *P < .05, **P < .01.

It has been well documented that susceptible (AKR) mice harbor the *T muris* worms until patency (day 35 after infection; Figure 1*A*), whereas the resistant (BALB/c) mice start expelling worms by day 14 after infection, and expulsion is achieved by day 21 after infection^22,36^ (Figure 1*A*). Changes in the production of Muc2, the main gel-forming constituent of intestinal mucus, were explored within the cecum of AKR or BALB/c mice exposed to a high-dose *T muris* infection. Immunohistochemical staining and reverse transcription–polymerase chain reaction (RT-PCR) analysis for Muc2 (Figure 1*B* and *C*) showed that significantly higher amounts of Muc2 were expressed within the cecal crypts of the resistant mice on day 21 after infection than in the naïve and susceptible mice; a similar staining pattern was observed with the PAS reagent (Supplementary Figure 1). This increase in goblet cell number and Muc2 levels was restricted to the niche of the parasite, was not observed in the colon (Supplementary Figure 1), and correlated with worm expulsion. Therefore, to further understand the role of Muc2 in *T muris* infection, we performed a high-dose infection in Muc2-deficient mice on the resistant C57BL/6 background.

### Muc2 Deficiency Delayed *T muris* Worm Expulsion From Infected Mice

**Figure 2:**
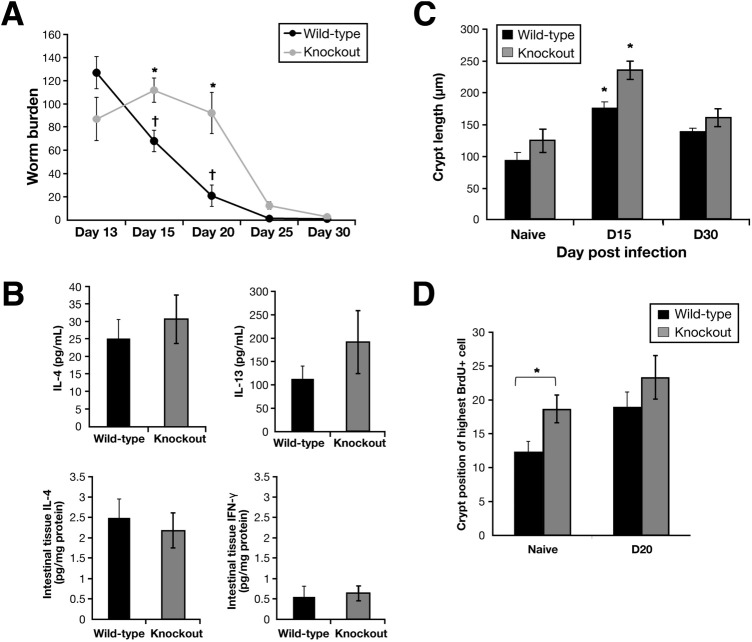
Muc2-deficient mice and their resistant WT (C57BL/6 background) littermates were infected orally with 300 eggs of *T muris*, and worm burdens were investigated on days 13, 15, 20, 25, and 30 after infection (*A*). Cytokine levels were determined in intestinal tissues (in pg/mg) or by concanavalin A stimulation of spleen cells (in pg/mL) (*B*). Cecal crypt length was measured (*C*), and crypt position of the highest BrdU^+^ cell (*D*) in Muc2-deficient and WT mice was determined. Representative of 5 mice. †*P* < .05 compared with day 13 after infection; *P < .05 compared with wild types. IFN-γ, interferon-γ.

A high-dose *T muris* infection established in both WT and Muc2-KO mice showed no marked difference in the number of worms at day 13 after infection (Figure 2*A*). However, as infection progressed, there was a significant decrease in worm burden in the WT mice, evident by day 15 after infection (46% reduction) and with a 84% decrease over establishment levels by day 20 after infection. In contrast, in the Muc2-deficient mice there was no decrease in worm burdens until after day 20 after infection, although mice did eventually expel their parasites.

### Muc2 Deficiency Had No Significant Effect on T_H_2-Type Immune Response Elicited by *T muris* Infection

We next sought to determine whether the delay in worm expulsion in the KO mice was due to an alteration to the adaptive immune response to *T muris* infection. Interleukin-4 (IL-4) and interferon-γ levels in intestinal tissue were not detectable in the naïve, WT, and KO mice. Furthermore, there was no significant difference in IL-4 or interferon-γ levels in intestinal tissues between both strains on day 20 after infection (Figure 2*B*). Consistent with the local immune response, there was no significant difference in IL-4 and IL-13 production from in vitro concanavalin A–stimulated spleen cells (Figure 2*B*). Thus, despite the delay in worm expulsion, Muc2 deficiency had no significant effect on generation of the T_H_2-type immune response in *T muris* infection. The crypt architecture, an indicator of inflammation, changed during infection; there was an increase in crypt length on day 15 after infection in WT and KO mice which was more pronounced in the KO mice (Figure 2*C*). With the use of the highest position of BrdU-positive cells in the crypts as a measure of rate of epithelial cell turnover,^33^ it was clear that cell turnover was higher in the naïve KO mice than in the WT mice. However, there was no significant difference in epithelial cell turnover between the KO and WT mice on day 20 after infection (Figure 2*D*), indicating that the delay in worm expulsion in KO mice was not associated with an alteration of the “epithelial escalator.”^37^

### *T muris* Infection Induced Expression of PAS-Positive Goblet Cells in Muc2-Deficient Mice

**Supplementary Figure 2:**
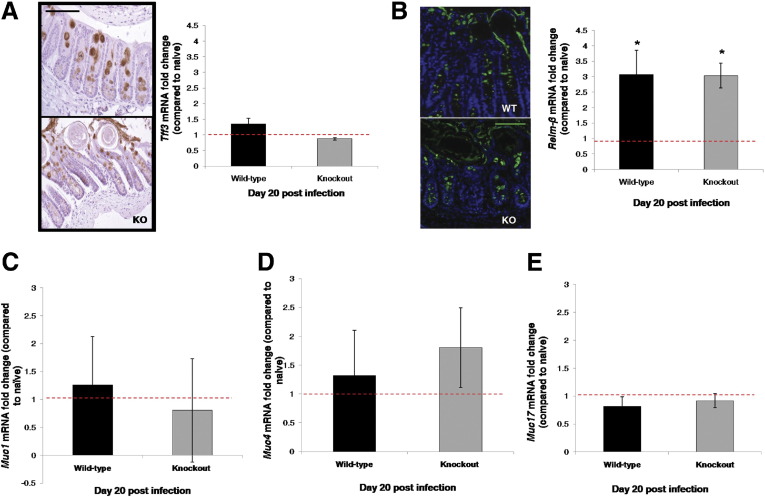
Expression of Tff3 (*A*) and Relm-β (*B*) were determined with the use of immunohistochemistry and RT-PCR in cecal tissue of Muc2 KO mice, and their resistant WT littermates on day 15 and day 20 after infection, respectively. RT-PCR showed no main changes in the mRNA expression of cell surface mucins, *Muc1* (*C*), *Muc4* (*D*), or *Muc17* (*E*) in the WT and KO mice on day 20 after infection. *Red dashed lines* indicate naïve levels. Scale bar, 10 μm. Representative of 5 mice. *P < .05.

**Supplementary Figure 3:**
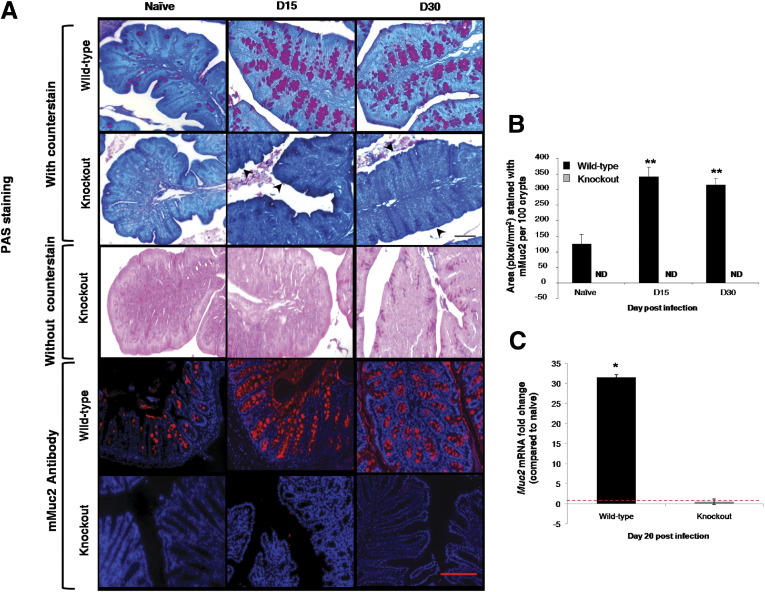
PAS staining with and without fast green counterstaining, and immunofluorescent staining with mMuc2 antibody of cecal tissue of Muc2 KO mice and their resistant WT littermates (*A*). *Arrows* highlight the emergence of smaller PAS-positive goblet cells in the Muc2 KO mice. Quantification of mMuc2 antibody staining represented as area stained in pixels per mm^2^ (*B*). RT-PCR confirms the increase in *Muc2* levels after infection in resistant mice (*C*; *red dashed line* indicates naïve levels). Representative of 5 mice. *P < .05.

**Figure 3:**
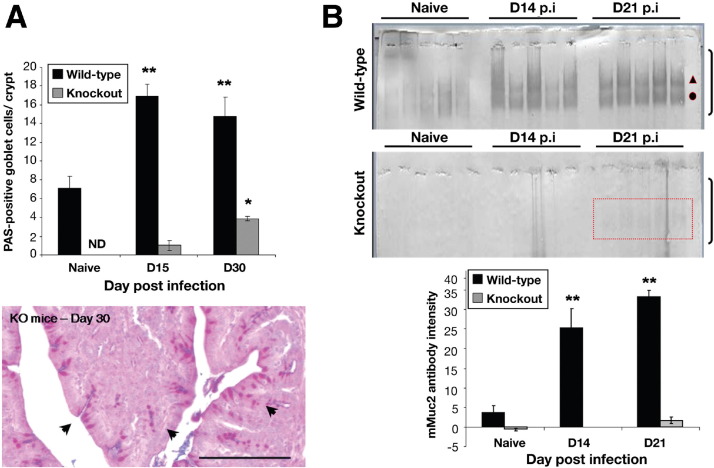
Quantification of goblet cell numbers in the cecum of WT and Muc2-deficient mice during infection (*A*); goblet cells marked by *arrows* in deficient mice can be visualized on day 30 after infection (PAS staining without fast green counterstain). Total mucus scraped from WT and Muc2-deficient mice were reduced/alkylated, separated by agarose gel electrophoresis, analyzed by Western blot, and probed with the mMuc2 antibody (*B*). The relative staining intensity of the mMuc2 antibody in the portion of the blot indicated by *brackets* was measured. A faint band (*red box highlighted*) was observed on day 21 after infection in the Muc2-deficient mice. The 2 Muc2 bands in the WT animals most likely represent the monomeric (●) and dimeric (▲) forms of Muc2 (*B*). Representative of 5 mice. *P < .05, **P < .01. ND indicates not detectable.

Despite the similar number of goblet cells (as defined by Relm-β and Tff3; Supplementary Figure 2) in the infected and noninfected KO and WT mice, there was a significant difference between the number of PAS-positive goblet cells (Figure 3*A*). As with the resistant BALB/c mice, there was a significant increase in the numbers of PAS-positive goblet cells in WT mice after infection. Although there was significant impairment in the development of hyperplastic goblet cells in the KO mice, unexpectedly by day 15 after infection, there was an increase in PAS-positive goblet cells, with significant elevation by day 30 after infection (Figure 3*A*; Supplementary Figure 3).

### *T muris* Infection Triggers Muc5ac Mucin Production

**Figure 4:**
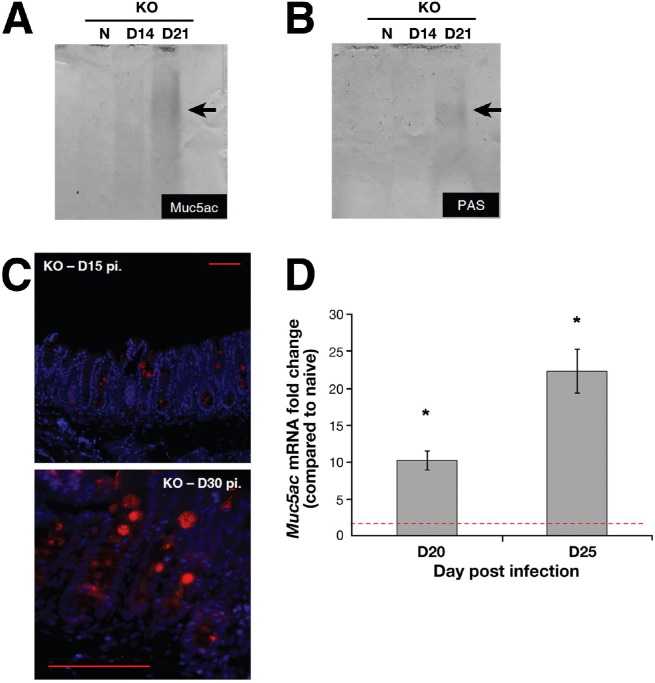
Muc5ac (*A*) and total glycoprotein (*B*) levels present in cecal mucus, determined by Western blotting using 45M1 antibody and PAS staining, respectively, in the Muc2-deficient mice. Immunofluorescence microscopy (*C*) and RT-PCR (*D*) illustrated Muc5ac was present in the Muc2-deficient mice after infection. D; *Red dashed line* = naïve levels. Representative of 5 mice. Scale bar; 10 μm. *P < .05, **P < .01.

After exposure to *T muris* the levels of Muc2 were significantly elevated in the WT mice (Supplementary Figure 3). As expected, no Muc2-positive goblet cells were seen in the KO mice. Similarly, higher amounts of Muc2 (assessed by Western blotting after agarose gel electrophoresis) were present in the content of mucus collected in the WT mice after infection (Figure 3*B*). Although there was little evidence of mature, glycosylated Muc2 in the KO mice, interestingly, on day 21 after infection, there was a faint band consistent with the electrophoretic migration of Muc2 in these mice (*red box*; Figure 3*B*). This, along with the PAS-positive goblet cells, suggested the presence of another polymeric mucin after infection. To identify this mucin, the mucus (pooled from 5 KO mice) was analyzed by Western blotting after agarose gel electrophoresis. A mouse Muc5b-antiserum did not show any bands (data not shown). In contrast, a Muc5ac monoclonal antibody^31,38^ identified bands in the mucus samples from infected mice (Figure 4*A*). Immunofluorescence microscopy (Figure 4*C*), RT-PCR (Figure 4*D*), and tandem mass spectrometry (data not shown) confirmed the de novo expression of Muc5ac after infection in the KO mice. Furthermore, the PAS-stained material after agarose gel electrophoresis showed coincidence with the Muc5ac reactive band (Figure 4*B arrow*), suggesting that Muc5ac is a significant component of the mucus in the KO animals. No marked changes were observed in the expression of the cell surface mucins, *Muc1*, *Muc4*, and *Muc17*, which are thought to contribute to mucosal protection (Supplementary Figure 3).

### Muc5ac Is Up-Regulated As Part of the “Normal” Response to Worm Expulsion

**Figure 5:**
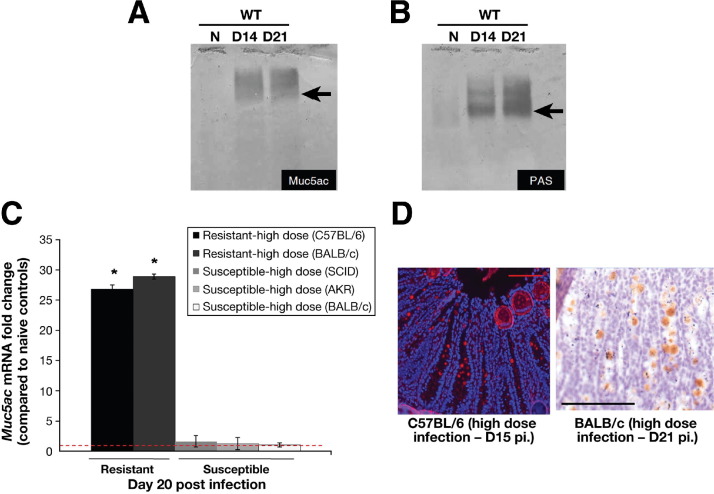
Muc5ac (*A*) and total glycoprotein (*B*) levels present in cecal mucus, determined by Western blotting with the use of 45M1 antibody and PAS staining, respectively, in the WT resistant (C57BL/6) mice. RT-PCR showed that *Muc5ac* levels increase significantly only in the resistant models (high-dose infection in BALB/c and C57BL/6 mice) and not in the susceptible models (low-dose infection in BALB/c and high-dose infection in AKR and SCID mice) (*C*; *red dashed line* indicates naïve levels). Immunofluorescence microscopy and immunohistochemistry showed that Muc5ac was present in some of the goblet cells of resistant mice after infection (*D*). Representative of 5 mice. Scale bar, 10 μm. *P < .05.

Unexpectedly, Muc5ac expression was also significantly up-regulated in the WT mice on days 14 and 21 after infection (Figure 5). In contrast to the KO mice, Western blotting showed that Muc5ac mucin was not the main component in the mucus, because the main PAS bands migrated further than the broad, Muc5ac-reactive band (Figure 5*arrow*) and was coincident with Muc2 staining bands (data not shown). However, the de novo expression of *Muc5ac* was only observed in the resistant mouse models (high dose in C57BL/6 and BALB/c mice) and not in the susceptible models (low dose in BALB/c, high dose in AKR and SCID mice) (Figure 5*C*). Immunofluorescence microscopy and immunohistochemistry confirmed the expression of Muc5ac after infection (days 15 and 21) in the cecal crypts of the resistant models (Figure 5*D*). No reactivity was observed in the susceptible models (data not shown).

### Susceptibility Is Associated With Altered Mucus Porosity

**Figure 6:**
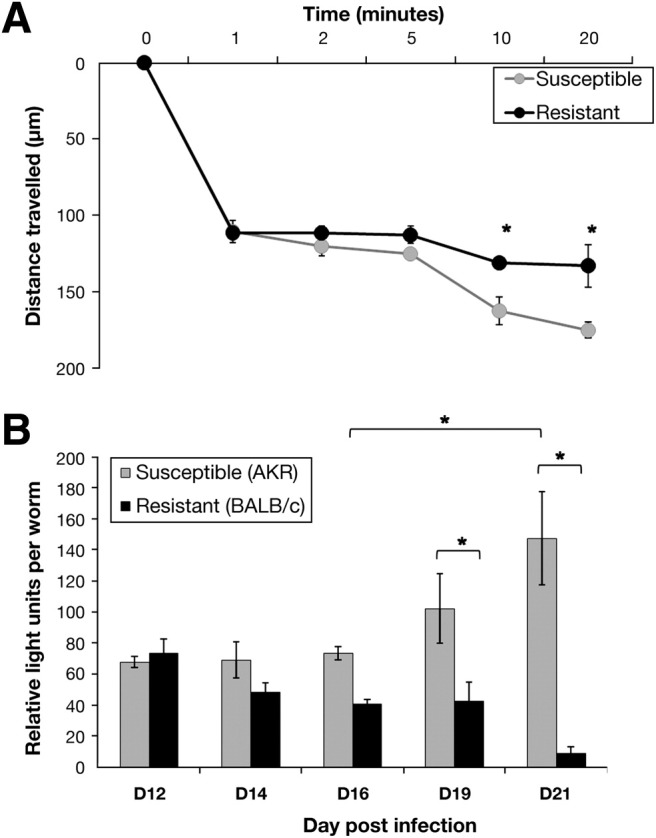
Fluorescent beads were used to determine the permeability of the mucus barrier of the susceptible (AKR) and resistant (BALB/c) mice on day 19 after infection, represented as the distance traveled from the top of the mucus barrier in the time stated (*A*). Energy levels (data presented as relative light units per worm) were determined in worms extracted from BALB/c and AKR mice during infection (*B*).

Fluorescently labeled beads were used to investigate mucus permeability after infection (day 19) in BALB/c and AKR mice (Figure 6*A*). The beads traveled to a depth of approximately 100 μm over a 60-second period in both strains. Thereafter, there was a reduction in diffusion rate of the beads in the resistant (BALB/c) mice. However, the beads traveled significantly further in the mucus of susceptible (AKR) mice over a 20-minute period.

### Worms in a Resistant Environment Have a Reduced Energy Status

**Supplementary Figure 4:**
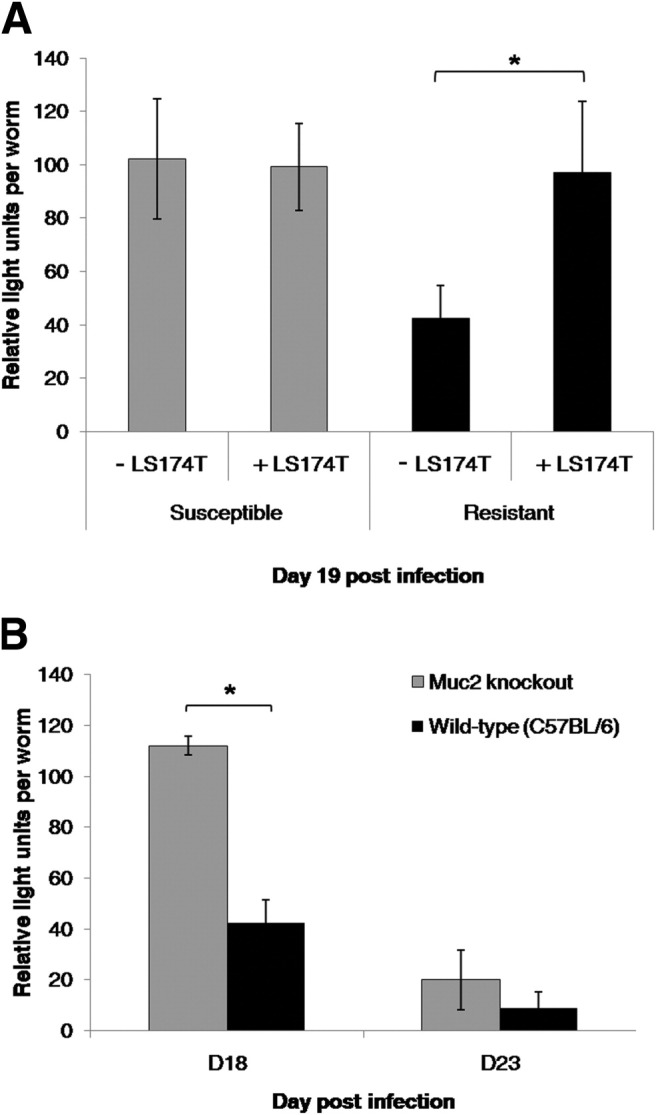
(*A*) ATP production (data presented as relative light units per worm) was determined in the worms isolated from the resistant (BALB/c) or susceptible (AKR) mice compared with isolated worms transferred onto LS174T cell culture on day 19 after infection. (*B*) ATP production by worms isolated from Muc2-deficient mice and their WT littermates was determined on days 18 and 23 after infection. Representative of 3 mice. *P < .05.

ATP measurements were carried out to determine the energy status of worms in resistant and susceptible mice as a measure of worm vitality. As infection progressed (day 21 after infection) in the AKR mice, there was a significant increase in the ATP production by the worms (Figure 6*B*). In contrast, there was a marked reduction in ATP production in the worms isolated from the BALB/c mice. However, these worms were not irreversibly damaged because they recovered their ATP production when transferred to in vitro culture with the colonic LS174T cell line for 24 hours (Supplementary Figure 4). Importantly, worms taken from Muc2-deficient mice showed a comparable drop in energy status during worm expulsion (Supplementary Figure 4).

## Discussion

It is well established that *T muris* survives by eliciting a T_H_1 response in mice susceptible to chronic infection in the absence of a T_H_2 response. In common with all other studies of intestinal helminth immunity, multiple effectors under immunologic (T_H_2) control are probably operating during worm expulsion. Although we already know that IL-13–mediated regulation of epithelial cell turnover and smooth muscle contractility can contribute to worm expulsion, little detail is known about the protective role of the secreted barrier, ie, mucus.^22,24,39^ Previously, we have shown that the T_H_2-type immune response in resistance plays an important role in the development of goblet cell hyperplasia.^14,40^ Some reports have also suggested that mucus produced from goblet cells has an important role in trapping and removing nematodes from the intestine.^17,18,20^ The polymeric mucins are responsible for the physical properties of the mucus barrier,^41,42^ and changes in mucins are associated with pathophysiology of a number of gastrointestinal disorders.^6,12^ It has also been shown that deficiency in the main component of the intestinal mucus barrier, Muc2, leads to an abnormal morphology of the colon and contributes to the onset and perpetuation of dextran sulfate sodium–induced experimental colitis.^6,11^

In this study we demonstrated, using the *T muris* model, that Muc2 increased in resistance (restricted to the cecum, the niche of the parasite) which correlated with worm expulsion. However, this was not the case for the mice susceptible (AKR) to *T muris* infection, supporting the hypothesis that Muc2 contributed to host protection in nematode infection. A distinct functional role for Muc2 in host protective immunity in *T muris* infection was shown in the Muc2-deficient mice. These animals exhibited a significant delay in worm expulsion even though the adaptive immune response was unaltered; similar T_H_2-type immune responses were shown in Muc2-deficient and WT control mice after infection. Unexpectedly, de novo expression of Muc5ac was observed just before worm expulsion in the Muc2 KO mice and resistant mouse models, but not in the susceptible models. Overall, the network properties of the intestinal mucus barrier are different between resistance and susceptibility, and the changes in the parasitic niche can have damaging effects on the vitality of the parasite. To our knowledge this is the first direct demonstration for a functionally protective role of gel-forming mucins in nematode infection.

Analysis of cecal mucus from Muc2-deficient mice showed that Muc5ac was the only polymeric mucin present in the mucus after infection. Moreover, in WT mice, although not the main mucin (which is Muc2), for the first time in a nematode infection we show the up-regulation of Muc5ac after intestinal infection. Several studies have elucidated that T_H_2-type cytokines such as IL-13 have the ability to up-regulate MUC5AC/Muc5ac expression levels.^43,44^ Therefore, the up-regulation in Muc5ac expression observed after infection, in both WT and Muc2-deficient mice, may be a result of IL-13 production. Interestingly, this de novo expression of Muc5ac was observed in all the resistant models (T_H_2-type response) but in none of the susceptible models (T_H_1-type response) of *T muris* infection. Although this mucin is predominantly found in airway and stomach mucus,^42,45^ studies on patients with ulcerative colitis and adenocarcinomas have shown MUC5AC expression in the intestine along with MUC2.^31,46^ However, this is the first time that Muc5ac expression has been implicated in response to an enteric parasitic infection.

We observed no discernible PAS-positive goblet cells throughout the cecum of Muc2-deficient mice without infection. However, this was not due to the absence of goblet cell lineage as the expression of Tff3, and Relm-β was observed in the cecum of both infected and noninfected WT and Muc2-deficient mice. This observation corroborates with the findings of Van der Sluis et al^6^ whereby the expression of Tff3 was observed, despite the lack of PAS-positive goblet cells. Muc2 seems to be the main phenotypic determinant of goblet cells, and, in the absence of Muc2, goblet cells lose their characteristic goblet-like shape and specific staining, but the goblet cell lineage is still present.^6,11^ Interestingly, after infection there was an increase in PAS-positive goblet cells in the Muc2-deficient mice. Although the size of the goblet cells in Muc2-deficient mice was smaller than in those in WT mice, their emergence correlated with worm expulsion.

We have shown a functional role for the mucus barrier in host protective immunity to *T muris* infection because in the absence of Muc2, worm expulsion is significantly delayed. Moreover, the physical properties of the mucus barrier are changed after infection, although the details of how these changes contribute to protection remains to be fully elucidated. However, one possibility is that, in the susceptible mice, the lower levels of Muc2 result in a network that may compromise defense because of inappropriate presentation or concentration of other host defense proteins in the environment of the worms. Whereas, in the resistant mice, other proteins (such as Relm-β, Tff3, and angiogenin) may be retained and effectively concentrated at the sites of worm infection. This may be by specific interactions with Muc2 or with the infected induced Muc5ac, or by the physical constraints imposed by the mucin network, thus rendering the host interface unsuitable for worm reproduction and/or survival which results in expulsion.^19^ Indeed, changes in the niche of the parasite do have a detrimental effect on the parasite, because worms extracted from mice during worm expulsion clearly have a reduced energy status than worms extracted from the susceptible mice. This reduction in the worm vitality is reversible if worms are transferred to a “favorable” environment, supporting the notion that expulsion reflects damaged, but not killed parasites.

Another explanation supported by our finding, which is by no means mutually exclusive, is that the physical nature of the mucus barrier is changed in such a way as to facilitate worm expulsion. We have shown that around the time of worm expulsion the mucus barrier is less porous in the resistant mice than in the susceptible mice, and this alteration in physical properties of the barrier after infection may directly affect the niche of the worms. The intestinal mucus barrier is proposed to comprise “loose” outer layer and a less porous, adherent inner layer.^7^ The results from the bead penetration assay showed that after 60 seconds the beads travelled to a depth of approximately 100 μm in the mucus from the susceptible and resistant mice, suggesting the properties of the loose layer are similar in both. However, the beads traveled at different rates thereafter, suggesting that the differences in network properties observed between the resistant and susceptible mice are mainly in the inner adherent layer of the barrier. This alteration may physically constrain the worms, thus affecting the niche.

What might be the role of the infection-induced mucin Muc5ac in protection against the worms? Muc5ac is assembled in a different manner to Muc2 and does not possess the disulphide-resistant cross-links present in Muc2^47–50^ and may result in a mucus gel with different rheologic properties. Indeed, Muc5ac is a main component of airway mucus, and, unlike the intestinal barrier which is normally an adherent Muc2-rich gel, a specific functional requirement in the airways makes a transportable mucus gel. Thus, Muc5ac may change the rheologic nature of the mucus gel and, in conjunction with the intestinal muscle hypercontractility (controlled by T_H_2 response^14,40^), could physically aid worm expulsion. This is consistent with the observations of mucus trapping in *N. brasiliensis* and *T. spiralis* infection, in which globules of mucus trap worms, which are then transported out of the intestine.^18,20^ Another interesting possibility raised by the data is that expulsion occurs in 2 phases: an early phase influenced by Muc2 and a final, clearance phase that occurs independently of Muc2, possibly involving Muc5ac.

In conclusion, this study clearly shows that the mucus barrier is a significant component of a well-coordinated response in the gut to worm expulsion. Even though *T muris* has an intracellular niche within the gut epithelium, in resistance as the “epithelial escalator” displaces worms, it may be that the overall changes in barrier have a subsequent significant detrimental effect on the worm itself, and the additional changes in the physical properties of mucus contribute to the efficient elimination of the worms from the intestinal lumen. Moreover, it further highlights the functionally dynamic and highly regulated nature of the mucus barrier during immunologically mediated intestinal disease.

## Supplementary Methods

### RT-PCR

Total RNA from epithelial cells was isolated with the use of the previously described method.^27^ cDNA was generated with the use of an IMPROM-RT kit (Promega) and Absolute QPCR SYBR Green (ABgene Epsom, Surrey, United Kingdom) was used for quantitative PCR. Primer efficiencies were determined with the use of cDNA dilutions, and genes of interest were normalized against housekeeping gene, β-actin, and expressed as a fold difference to uninfected naïve message levels. mRNA expression was investigated with the primers 5′-GTGGGCCGCTCTAGGCACCAA-3′ and 5′-CTCTTTGATGTCACGCACGATTTC-3′ for β-actin, 5′-GTCCAGGGTCTGGATCACA- 3′ and 5′-CAGATGGCAGTGAGCTGAGC-3′ for Muc2; 5′-GTGATGCACCCAT GATCTATTTTG-3′ and 5′-ACTCGGAGCTATAACAGGTCATGTC-3′ for Muc5ac, GGTTGCTTTGGCTATCGTCTATTT and AAAGATGTCCAGCTGCCCATA for Muc1, CCACCTCCTCGACCCTTACT and CTCCGACTTCAGACCCGTAG for Muc4, 5′-GTGGGACGGGCTCAAATG-3′ and 5′-CTC TACGCTCTCCACCAGTTCCT-3′ for Muc17, 5′-TTGCTGGGTCCTCTGGGATA-3′ and 5′-GCCGGCACCATA CATTGG-3′ for Tff3, and 5′-GCTCTTCCCTTTCCTTCTCCAA-3′ and 5′-ACCACAGTGTAGGCTTCATGCTGTA-3′ for Relm-β. RT-PCR products were directly sequenced to verify the identity of the amplified genes. In brief, products were digested with Exonuclease I and calf intestinal phosphatase and subsequently sequenced with the use of the ABIPRISM (Applied Biosystems, Foster City, CA) Big-Dye Terminator cycle sequencing reaction at the Sequencing Facility in the University of Manchester. The data were analyzed with Chromas Pro v1.34 (Technelysium P/L, Tewantin, QLD, Australia), and the sequences obtained were compared against the GenBank database (http://www.ncbi.nlm.nih.gov/BLAST).

### Rate of Epithelial Cell Turnover

The rate of intestinal epithelial cell turnover was assessed by visualizing BrdU incorporated into nuclei after mice were injected with 10 mg of BrdU 16 hours before killing, as described previously.^37^

### Evaluation of In Vitro Cytokines Production From Splenocytes

Single-cell suspensions of spleen were prepared in RPMI 1640 containing 10% fetal calf serum, 5 mmol/L l-glutamine, 100 U/mL penicillin, 100 mg/mL streptomycin, 25 μm/L HEPES, 0.05 mmol/L 2-ME (all from Gibco-BRL, Carlsbad, CA). Cells (10^7^) were incubated in the presence of 5 mg/mL concavalin A (Con A). IL-4 and IL-13 levels in the supernatant were measured by enzyme immunoassay with the use of a commercially available kit (R&D Systems, Minneapolis, MN).

### Investigation of Intestinal Tissue Cytokine Levels

Frozen intestinal tissues were homogenized in lysis buffer containing protease inhibitor cocktail (Sigma, Indianapolis, IN). The homogenates were freeze-thawed 3 times and centrifuged, and then supernatant was collected and stored at −20°C until analyzed. Interferon-γ, IL-4, and IL-13 levels in the supernatant were measured by enzyme immunoassay technique with the use of a commercially available kit purchased from R&D Systems. Concentration of protein in the intestinal tissue was determined by a commercially available DC Protein Assay kit (Bio-Rad, Hercules, CA), and the amount of cytokines in the tissues was expressed per milligram of tissue protein.

### Quantification of Histologic Staining

The numbers of goblet cells expressed per crypt were counted in 50 longitudinally sectioned crypt units. The area stained (pixel/mm^2^) per 100 crypts was determined by using the ImageJ software Version 1.39a (National Institutes of Health, Bethesda, MD).

### Worm Isolation for ATP Analysis

The cecum was longitudinally cut and segmented before incubation with 0.1 mol/L NaCl for 2 hours at 37°C with frequent shaking. Worms were counted after separation from debris and epithelial cells with the use of a 0.7-μm filter and kept in RPMI 1640 supplemented with 10% fetal calf serum.. Alive worms were subsequently homogenized with the use of the FastPrep homogeniser (MP Biomedicals, Irvine, CA).
